# Potential Phosphorylation of Viral Nonstructural Protein 1 in Dengue Virus Infection

**DOI:** 10.3390/v13071393

**Published:** 2021-07-17

**Authors:** Thanyaporn Dechtawewat, Sittiruk Roytrakul, Yodying Yingchutrakul, Sawanya Charoenlappanit, Bunpote Siridechadilok, Thawornchai Limjindaporn, Arunothai Mangkang, Tanapan Prommool, Chunya Puttikhunt, Pucharee Songprakhon, Kessiri Kongmanas, Nuttapong Kaewjew, Panisadee Avirutnan, Pa-thai Yenchitsomanus, Prida Malasit, Sansanee Noisakran

**Affiliations:** 1 Division of Molecular Medicine, Research Department, Faculty of Medicine Siriraj Hospital, Mahidol University, Bangkok 10700, Thailand; thanyaporn.dec@mahidol.ac.th (T.D.); pucharee.son@mahidol.ac.th (P.S.); ptyench@gmail.com (P.-t.Y.); 2 Functional Proteomics Technology Laboratory, Functional Ingredients and Food Innovation Research Group, National Center for Genetic Engineering and Biotechnology, National Science and Technology Development Agency, Bangkok 12120, Thailand; sittiruk@biotec.or.th (S.R.); y.yingchutrakul@gmail.com (Y.Y.); sawanya2010@gmail.com (S.C.); 3 Molecular Biology of Dengue and Flaviviruses Research Team, Medical Molecular Biotechnology Research Group, National Center for Genetic Engineering and Biotechnology, National Science and Technology Development Agency, Bangkok 10700, Thailand; bunpote.sir@biotec.or.th (B.S.); arunothai.duck@gmail.com (A.M.); tanapan.pro@biotec.or.th (T.P.); chunyapk@biotec.or.th (C.P.); prida.mal@mahidol.ac.th (P.M.); 4 Division of Dengue Hemorrhagic Fever Research, Research Department, Faculty of Medicine Siriraj Hospital, Mahidol University, Bangkok 10700, Thailand; kessiri.kon@mahidol.ac.th (K.K.); nuttapong.kae@mahidol.ac.th (N.K.); panisadee.avi@mahidol.edu (P.A.); 5 Department of Anatomy, Faculty of Medicine Siriraj Hospital, Mahidol University, Bangkok 10700, Thailand; thawornchai.lim@mahidol.ac.th; 6 Siriraj Center of Research Excellence in Dengue and Emerging Pathogens, Faculty of Medicine Siriraj Hospital, Mahidol University, Bangkok 10700, Thailand

**Keywords:** dengue virus, NS1, phosphorylation, LC-MS/MS, virus production

## Abstract

Dengue virus (DENV) infection causes a spectrum of dengue diseases that have unclear underlying mechanisms. Nonstructural protein 1 (NS1) is a multifunctional protein of DENV that is involved in DENV infection and dengue pathogenesis. This study investigated the potential post-translational modification of DENV NS1 by phosphorylation following DENV infection. Using liquid chromatography-tandem mass spectrometry (LC-MS/MS), 24 potential phosphorylation sites were identified in both cell-associated and extracellular NS1 proteins from three different cell lines infected with DENV. Cell-free kinase assays also demonstrated kinase activity in purified preparations of DENV NS1 proteins. Further studies were conducted to determine the roles of specific phosphorylation sites on NS1 proteins by site-directed mutagenesis with alanine substitution. The T27A and Y32A mutations had a deleterious effect on DENV infectivity. The T29A, T230A, and S233A mutations significantly decreased the production of infectious DENV but did not affect relative levels of intracellular DENV NS1 expression or NS1 secretion. Only the T230A mutation led to a significant reduction of detectable DENV NS1 dimers in virus-infected cells; however, none of the mutations interfered with DENV NS1 oligomeric formation. These findings highlight the importance of DENV NS1 phosphorylation that may pave the way for future target-specific antiviral drug design.

## 1. Introduction

Dengue virus (DENV) is the etiologic agent of the mosquito-borne diseases dengue fever, dengue hemorrhagic fever, and dengue shock syndrome, which globally affect 67–136 million people each year [1,2]. Infected individuals experience varying degrees of clinical manifestations that are caused by unclear underlying pathogenic mechanisms [3]. At present, there is neither a specific antiviral drug for treatment of dengue nor a predictive marker of disease severity. A licensed dengue vaccine is currently available in some countries with specific indications, whereas the development of other dengue vaccine formulations is still in progress to seek for better disease protection and no possible risk of disease enhancement [4,5,6,7,8]. 

DENV is an enveloped, positive-sense, single-stranded RNA virus in the genus *Flavivirus* of the family *Flaviviridae* that includes four antigenically distinct serotypes [9]. Infection of target cells with DENV results in the production of three viral structural proteins (capsid, C; pre-membrane, prM; and envelope, E) that are required for virion formation, and seven viral nonstructural proteins (NS1, NS2A, NS2B, NS3, NS4A, NS4B, and NS5) that play important roles in viral polyprotein processing and viral RNA replication [10]. Of all of the DENV proteins, NS1 appears to be the viral protein that is detectable in the blood circulation of infected individuals at levels likely related to disease severity, so it is an important diagnostic marker of DENV infection [11,12,13]. The NS1 protein is newly synthesized in the lumen of the endoplasmic reticulum (ER) as a monomer and subsequently becomes a homodimer with post-translational modifications, including N-linked glycosylation, glycosylphosphatidylinositol linkage, and lipid raft association [14,15,16,17,18]. The DENV NS1 protein is further transported and secreted into the extracellular milieu as a hexameric lipoprotein [19,20]. In addition, the NS1 protein can be detected on the surface of virus-infected cells either through its membrane association or by binding to specific sulfated glycosaminoglycans [14,15,21]. 

The DENV NS1 protein plays different roles in the production of infectious virus, immune evasion, and dengue pathogenesis. Intracellular NS1 colocalizes with double-stranded viral RNA in virus-induced membrane structures (vesicle packets), which most likely act as the sites of viral RNA replication [22,23,24]. NS1 interaction with the DENV NS4A-2K-4B precursor is required for viral RNA replication [25]. The trans-complementation of NS1 restores viral RNA replication in defective DENV and other related flaviviruses [26,27,28], which suggests its function as a cofactor in flavivirus replication. NS1 also associates with DENV structural proteins and modulates the production of infectious DENV particles [28]. The secreted NS1 protein supports DENV infection by enhancement of virus production in target cells [29,30] and by inhibition of complement-mediated virus neutralization [31,32,33]. The soluble NS1 protein also induces immune cells via Toll-like receptor binding to release pro-inflammatory cytokines, it triggers endothelial cell hyperpermeability, and it participates in immune complex formation and subsequent complement activation, which may lead to pathogenic mechanisms of dengue [34,35,36,37,38,39,40]. Cross-linking of membrane-associated NS1 on the cell surface with specific antibodies results in the deposition of complement components on virus-infected cells [39] and induction of intracellular tyrosine phosphorylation [14]. Taken together, this evidence suggests the involvement of the DENV NS1 protein in virus replication and host cellular events in response to DENV infection.

Protein phosphorylation is an important post-translational modification that regulates functions of cellular proteins in eukaryotic cells [41,42,43]. Infection with DENV has been demonstrated to induce the activation of intracellular signaling proteins in the mitogen-activated protein kinase (MAPK) pathways, including the two extracellular signal-regulated kinases (ERK1/2), the Jun N-terminal kinase (JNK), and p38—all of which participate in viral replication and pathogenesis [44,45]. Specifically, previous studies showed that the NS1 protein of DENV increases nuclear translocation and transcriptional activity of the NF-kB protein [46], activates the p38 MAPK pathway [40], and interacts with a number of host cellular proteins (some of which are involved in signal transduction, transcriptional and translational processes, protein folding and modifications, vesicle trafficking and protein transport, and cell metabolism and development) that might be modulated by protein phosphorylation [31,32,47,48,49,50,51,52,53,54]. Whether the DENV NS1 protein has additional post-translational modification related to host phosphorylation machinery following DENV infection needs to be addressed. In this study, we set forth to investigate potential phosphorylation sites on the DENV NS1 protein using immunoprecipitation with specific antibodies and liquid chromatography-tandem mass spectrometry (LC-MS/MS), and we determined the functional importance of identified specific amino acid residues relative to intrinsic properties of the NS1 protein and infectious DENV production. Our results revealed novel findings specific to the phosphorylation of DENV NS1, which exerts functional effects on virus amplification during DENV infection.

## 2. Materials and Methods

### 2.1. Cell Lines, Viruses, and Antibodies 

Hepatocellular carcinoma HepG2 (ATCC HB-8065) and Huh7 (JCRB0403, HuH-7) cells and baby hamster kidney fibroblast cells (BHK-21, ECACC 85011433) were cultured in supplemented 10% fetal bovine serum (FBS)–Dulbecco’s modified Eagle medium (DMEM) (Gibco; Invitrogen, Carlsbad, CA, USA) at 37 °C in a 5% CO_2_ humidified atmosphere, as previously described [53,55,56]. African green monkey kidney epithelial cells (Vero cells, ATCC CCL-81) were cultured in 10% FBS–minimum essential medium (MEM) (Gibco) in the presence of 2 mM L-glutamine, 36 µg/mL penicillin G, and 60 µg/mL streptomycin in the same culture conditions. Dengue virus serotype 2 (DENV-2, strain 16681) was propagated in mosquito C6/36 cells (provided by the Armed Forces Research Institute of Medical Sciences, Thailand). Mouse monoclonal antibodies specific for DENV NS1 (clones NS1-3F.1 and 1A4) and DENV E (clone 4G2) were produced from previously established hybridoma cells [57,58,59]. Mouse IgG1 and IgG2a isotype-matched antibodies were purchased from Sigma-Aldrich Corporation (St. Louis, MO, USA). Mouse anti-human glyceraldehyde 3-phosphate dehydrogenase (GAPDH) antibody, horseradish peroxidase-conjugated rabbit anti-mouse IgG antibody, and Alexa Fluor 488-conjugated goat anti-mouse IgG antibody were purchased from Santa Cruz Biotechnology (Dallas, TX, USA), Dako (Glostrup, Denmark), and Invitrogen Corporation (Carlsbad, CA, USA), respectively.

### 2.2. Purification of Cell-Associated and Extracellular NS1 Proteins

To prepare the cell-associated NS1 proteins, Huh7, Vero, and HepG2 cells were seeded in T-162 cm^2^ flasks (Costar; Corning, Inc., Corning, NY, USA), and on the next day, they were incubated with DENV-2 at a multiplicity of infection (MOI) of 0.5, 1, and 5, respectively, at 37 °C in a 5% CO_2_ incubator for 2 h. Thereafter, the supernatant was replaced with fresh culture medium, and the culture was maintained under the same condition. The virus-infected cells were harvested at 24 h for HepG2 and Vero cells and at 48 h for Huh7 cells after infection and subjected to immunoprecipitation assay using anti-NS1 antibodies or their isotype-matched control antibodies, as previously described [53]. The immunoprecipitated samples were quantitated for total protein concentration by Bradford Protein Assay Kit (Bio-Rad Laboratories, Hercules, CA, USA). Three independent sets of samples were prepared for each cell type. To prepare the extracellular NS1 proteins, the three cell lines were infected with DENV-2 in serum-free medium, and the culture supernatants were collected at day 2 for Huh7 and HepG2 cells and at day 8 for Vero cells after infection. The supernatants were sequentially centrifuged at 3000 revolutions per minute (rpm) for 10 min, 10,000 rpm for 1 h, and 45,000 rpm for 4 h at 4 °C to separate out the cell debris and DENV particles. Clear supernatants were subjected to affinity chromatography using cyanogen bromide-activated Sepharose 4B beads (GE Healthcare, Uppsala, Sweden) covalently coupled to an anti-DENV NS1 antibody. Extracellular NS1 proteins were eluted from the column with 20 mM diethylamine-phosphate-buffered saline (PBS) pH 11.3, neutralized with 100 mM Tris-HCl pH 6.0, and subjected to buffer exchange in 10 mM Tris-HCl pH 7.5. Concentrations of extracellular NS1 were quantified using a FluoroProfile Protein Quantification Kit (Sigma-Aldrich). The immunoprecipitated samples and purified extracellular NS1 proteins were analyzed by 10% sodium dodecyl sulfate-polyacrylamide gel electrophoresis (SDS-PAGE) and colloidal Coomassie Brilliant Blue G-250 staining. Protein bands corresponding to DENV NS1 were excised from the gels for further processing prior to LC-MS/MS analysis.

### 2.3. Mass Spectrometric Analysis of DENV NS1 Phosphopeptides

The excised gel fragments containing cell-associated or extracellular NS1 proteins (total approximately 20–25 µg) were subjected to dehydration in 100% acetonitrile and incubated at 56 °C for 1 h with 10 mM dithiothreitol (DTT) in 10 mM NH_4_HCO_3_ for disulfide bond reduction. Then, the gel fragments were incubated with 100 mM iodoacetamide in 10 mM NH_4_HCO_3_ in the dark at room temperature (RT) for 45 min for alkylation, followed by dehydration with 100% acetonitrile at RT for 5 min. Sequencing-grade trypsin (Promega Corporation, Madison, WI, USA) was added to the gel fragments that were then incubated at 37 °C overnight for in-gel protein digestion. Peptide products were extracted from the gel fragments by incubation with 50% acetonitrile in 0.1% formic acid at RT for 10 min and dried at 45 °C for 4 h. Tryptic peptides were protonated with 0.1% formic acid and prepared for injection into an Ultimate 3000 Nano/Capillary LC System (Thermo Fisher Scientific, Waltham, MA, USA) coupled to a Hybrid Quadrupole Q-Tof Impact II (Bruker Daltonics, Billerica, MA, USA) with a nano-captive spray ion source, as previously described [60]. Briefly, peptides were enriched in an Acclaim PepMap100 C18 column (5 μm, 100 Å, 300 μm i.d. × 5 mm; Thermo Fisher Scientific) and separated in an Acclaim PepMap RSLC C18 column (2 μm, 100 Å, nanoViper, 75 μm i.d. × 15 cm; Thermo Fisher Scientific). Solvents A and B containing 0.1% formic acid in water and 0.1% formic acid in 80% acetonitrile, respectively, were introduced into the analytical column. A gradient of 5–55% solvent B was used to elute the peptides at a constant flow rate of 0.30 μL/min for 30 min. Electrospray ionization was performed at 1.6 kV using a CaptiveSpray instrument (Bruker Daltonics). Mass spectra (MS) and tandem mass spectra (MS/MS) were obtained in positive-ion mode within a range of mass-to-charge ratio (*m*/*z*) of 150 to 2200 (Compass 1.9 software; Bruker Daltonics). The LC-MS data were submitted for a database search using Mascot software from Matrix Science, London, UK [61] with carbamidomethyl cysteine as fixed modification and with oxidized methionine and phosphorylation of serine, threonine, and tyrosine as variable modifications (allowing neutral loss of 98 for pS/T but not pY peptides). The mass tolerances of the precursor ions and the fragment ions were set at 1.2 Da and 0.6 Da, respectively. The data of matched peptides were searched against the DENV NS1 protein sequence. Phosphorylation sites on the peptide sequences were computed from Mascot search result files.

### 2.4. Kinase Assay of DENV NS1 Proteins

Kinase activity of DENV NS1 proteins was determined by ADP-Glo Kinase Assay (Promega) based on the luminescence-based detection of ADP. Briefly, purified extracellular NS1 proteins from DENV-infected HepG2, Huh7, and Vero cell cultures, or myelin basic protein (unrelated control protein; Promega) in the amount of 0.5 µg were incubated with kinase buffer containing 40 mM Tris pH 7.5, 20 mM MgCl_2_, 0.1 mg/mL BSA, and 50 µM DTT in the presence or absence of 10 µM ultra-pure ATP in a total volume of 5 µL in a 384-well plate (Corning) at 25 °C for 1 h. Reactions that contained kinase buffer alone in the presence or absence of 10 µM ultra-pure ATP were also set up in parallel to serve as baseline controls for kinase activity. Thereafter, the kinase reactions were incubated with 5 µL of ADP-Glo reagent (Promega) at 25 °C for 40 min to stop the kinase reaction and to deplete the unconsumed ATP. Then, the reactions were added with 10 µL of kinase detection reagent (Promega) and incubated at 25 °C for 30–60 min to facilitate the conversion of newly synthesized ADP to ATP, which is used for an enzymatic reaction between luciferase and luciferin substrate to generate luminescence signals. Luminescence was measured using a Synergy H1 multi-mode microplate reader (Biotek Instruments, Winooski, VT, USA) with an integration time of 50 milliseconds in each readout for a total 10 times. The mean reading for each reaction was used for analysis of kinase activity.

### 2.5. Construction of DENV with NS1 Phosphorylation Site Mutations

A plasmid comprising a pcDNA3.1 Hygro vector (Invitrogen) with a cloned DENV NS1-encoding gene was used as a template for site-directed mutagenesis with alanine substitution at potential phosphorylation sites (T27A, T29A, Y32A, T230A, and S233A) and at a non-phosphorylated site predicted for human GRP78 binding (V6A, unrelated control) of DENV NS1 according to a previously described method [55]. The oligonucleotide primers for site-directed mutagenesis are shown in Table S1. Wild-type and mutant DENV NS1 fragments were amplified from the generated plasmid constructs and verified for their nucleotide sequences by Sanger DNA sequencing (Macrogen, Seoul, South Korea). The purified DENV NS1 amplicons were subjected to Gibson assembly together with other DENV gene fragments and a plasmid expression vector, as previously described [62]. The resulting full-length DENV cDNAs were transfected into BHK-21 cell cultures using Lipofectamine 2000 (Invitrogen), and the culture supernatants were harvested on day 3 post-transfection and transferred into Vero cell cultures to propagate the infectious DENV. Supernatants from Vero cell cultures were collected at different time points post-infection and assessed for the presence of infectious DENV by dot enzyme immunoassay and focus-forming unit (FFU) assay using an anti-DENV E antibody [63]. DENV propagation was repeated once again in Vero cell cultures. DENV NS1 sequences of wild-type and mutant viruses were verified by Sanger DNA sequencing analysis (Macrogen).

### 2.6. Kinetic Studies of DENV with NS1 Phosphorylation Site Mutations

Vero cells were plated onto black 96-well plates (Corning) at 2.5 × 10^4^ cells/well and cultured for 24 h at 37 °C in a 5% CO_2_ incubator. Then, the cells were incubated under the same conditions for 2 h with wild-type or NS1 mutant viruses (T29A, T230A, S233A, and V6A) at an MOI of 0.1 in a total volume of 100 µL or with an equal volume of supernatant from T27A and Y32A mutant-infected Vero cell cultures whose infectious virus titers could not be determined by FFU assay. Vero cells that were incubated with medium alone (mock) served as a negative control for DENV infection. Thereafter, mock and virus-infected cells were washed twice with 200 µL of plain medium and maintained in 200 µL of complete medium under the same culture conditions. At different time points post-infection, culture supernatants were collected and assessed for DENV NS1 secretion and infectious virus production by NS1 enzyme-linked immunosorbent assay (ELISA) [64] and FFU assay [63], respectively. In parallel, mock and virus-infected cells were determined for percentage of DENV infection and relative expression of DENV NS1 protein by immunofluorescence staining assay (IFA).

### 2.7. Detection of DENV Infection and DENV NS1 Expression

At varying time points post-infection, the mock and DENV-infected Vero cells in black 96-well plates were washed with PBS, fixed with 4% paraformaldehyde in PBS for 20 min, and permeabilized with 0.2% Triton-X-100 in PBS for 10 min at RT. Thereafter, the cells were successively incubated at RT with a mouse anti-NS1 monoclonal antibody (clone 1A4) for 1 h and with a mixture of Alexa Fluor 488-conjugated goat anti-mouse IgG antibody (1:1000 dilution), Hoechst 33342 (1:1000 dilution; Molecular Probes, Eugene, OR, USA), and HCS CellMask Deep Red Stain (1:50,000 dilution; Invitrogen) for 30 min in the dark. Three washes with PBS were performed after the primary and secondary staining steps. Images of the stained cells were captured with a 20× objective lens for 25 fields in each sample and analyzed by an Operetta High Content Imaging System using Harmony high-content imaging and analysis software version 4.1 (PerkinElmer, Inc., Waltham, MA, USA) to assess the proportion of DENV-infected cells and the relative levels of DENV NS1 expression. 

### 2.8. Immunoblotting Analysis for DENV NS1 Formation 

To determine monomeric and dimeric formation of DENV NS1, mock and DENV-infected Vero cell lysates were prepared in the presence and absence of 5% β-mercaptoethanol and heat treatment at 95 °C for 5 min prior to 10% SDS-PAGE and immunoblotting analysis according to a previously reported method [63]. Mouse monoclonal antibodies specific for DENV NS1 and human glyceraldehyde 3-phosphate dehydrogenase (GAPDH, an internal protein control) were used for detection of the corresponding proteins. Relative levels of DENV NS1 expression were assessed by normalization of protein band intensities to human GAPDH using GeneTools software (Syngene, Cambridge, UK). To determine oligomeric formation of DENV NS1, the supernatants of DENV-infected Vero cell cultures were subjected to native PAGE and immunoblotting according to a previously described method with minor modifications [65].

### 2.9. Verification of DENV NS1 Phosphorylation Mutations by LC-MS/MS

Vero cells infected with DENV NS1 mutant viruses (T29A, T230A, and S233A) or wild-type DENV were processed for immunoprecipitation of DENV NS1 using a previously described method [53]. The immunoprecipitated samples were analyzed by 10% SDS-PAGE and colloidal Coomassie Brilliant Blue G-250 staining. DENV NS1 protein bands were excised from the gels and processed for LC-MS/MS analysis using the aforementioned protocol to search for peptides that contain the corresponding mutations at specific amino acid positions of DENV NS1 as compared to their wild-type counterpart. 

### 2.10. Statistical Analysis

Effects of DENV NS1 phosphorylation site mutations on DENV infection, DENV NS1 expression and secretion, and DENV production were statistically analyzed by comparison with their wild-type counterpart using GraphPad Prism version 9.0.0. (San Diego, CA, USA). Differences in the proportion of DENV-infected cells, the relative levels of cell-associated NS1 expression, the amounts of extracellular NS1 protein, and titers of infectious DENV production were compared by two-way analysis of variance (ANOVA) with Dunnett’s post hoc multiple comparisons test. Differences in relative levels of monomeric and dimeric DENV NS1 protein expression were compared by one-way ANOVA with Dunnett’s post hoc multiple comparisons test. Differences in relative luminescence units between DENV NS1 derived from each cell type and buffer control in the presence of ATP were analyzed by an unpaired t-test. A *p*-value less than 0.05 indicates a statistically significant difference between variables.

## 3. Results

### 3.1. Identification of Potential Phosphorylation Sites on the DENV NS1 Protein

**Figure 1:**
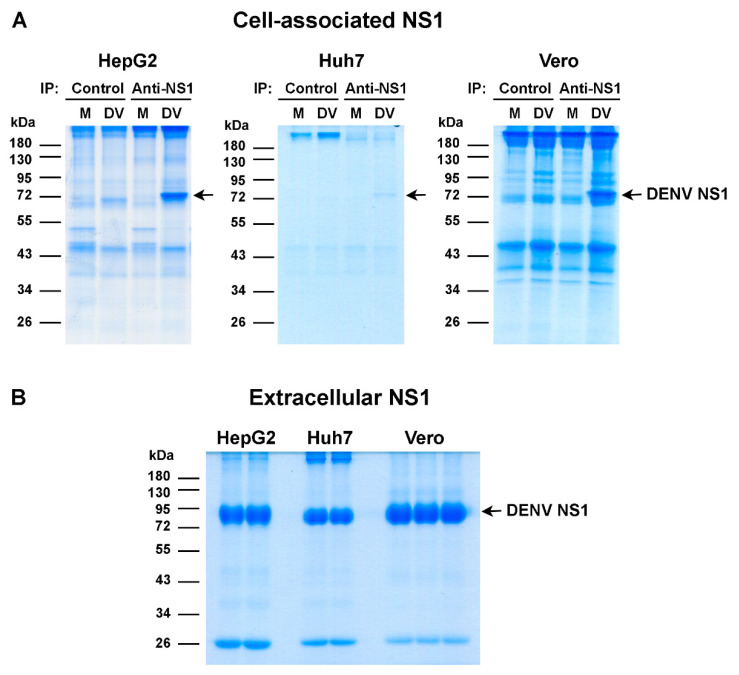
Preparation of cell-associated and extracellular NS1 proteins for LC-MS/MS analysis. HepG2, Huh7, and Vero cells were infected with DENV (DV) or left uninfected (mock, M). Cell lysates were harvested at day 2 post-infection and processed for immunoprecipitation with anti-NS1 antibodies (anti-NS1) or isotype-matched control antibodies (control). Culture supernatants were collected at day 2 (HepG2 and Huh7) or day 8 (Vero) post-infection and subjected to affinity chromatography to purify extracellular DENV NS1 using Sepharose 4B beads covalently linked with an anti-NS1 monoclonal antibody. Cell-associated NS1 proteins (**A**) and extracellular NS1 proteins (**B**) were analyzed by SDS-PAGE under non-heated and non-reduced conditions and Coomassie Brilliant Blue staining. Results show representative images of the stained gels. DENV NS1 protein bands (arrows; total approximately 20–25 µg) were excised from the gels and further processed for LC-MS/MS analysis of NS1 phosphopeptides.

Cell-associated and extracellular NS1 proteins were prepared from DENV-infected HepG2, Huh7, and Vero cell cultures by immunoprecipitation and affinity chromatography and subsequently analyzed by SDS-PAGE and Coomassie Brilliant Blue staining as described in the Materials and Methods section. Protein bands corresponding to DENV NS1 protein (approximately 80 kDa in a dimeric form) were specifically detected in DENV-infected cell lysates from all cell types immunoprecipitated with anti-NS1 antibodies as compared with those immunoprecipitated with isotype-matched control antibodies, or mock-infected cell lysates immunoprecipitated with either anti-NS1 antibodies or control antibodies (Figure 1A). In all cell types, extracellular NS1 proteins had slightly higher molecular weight bands than cell-associated NS1 proteins (Figure 1B).

**Figure 2:**
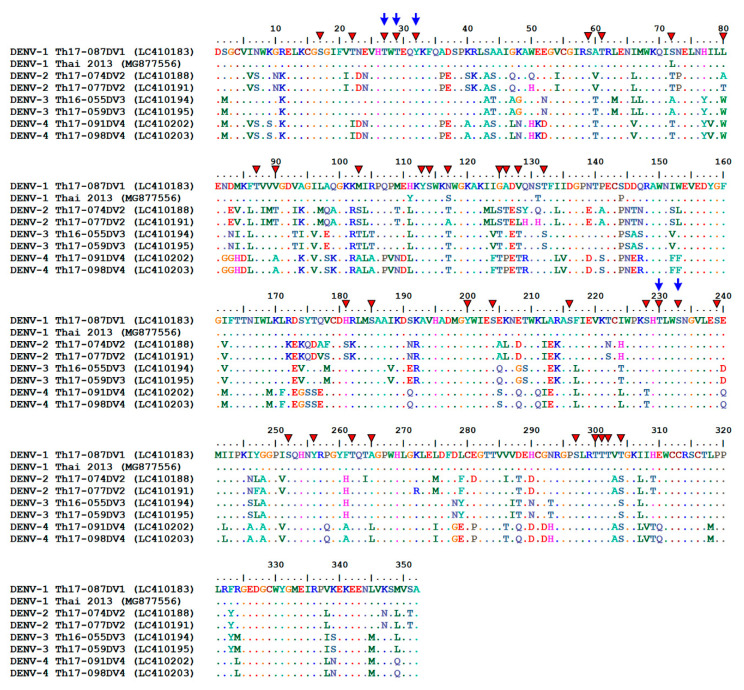
Amino acid sequence alignment of DENV NS1 proteins. Amino acid sequences of DENV NS1 were retrieved from the Virus Pathogen Database and Analysis Resource (ViPR) for dengue virus, which includes data specific to dengue virus isolation from humans in Thailand during 2000–2020. A total of 638 sequences of DENV NS1 (i.e., 330, 83, 105, and 120 sequences for virus serotypes 1, 2, 3, and 4, respectively) were obtained from the database and subjected to amino acid sequence alignment using BioEdit software version 7.2.5. The results show the alignment of two representative sequences of DENV NS1 (352 amino acid residues) from each serotype. Dots represent similar identity of amino acids at different positions on the NS1 protein. Potential phosphorylation sites at specific amino acid residues of DENV NS1, which were identified by LC-MS/MS, are shown in red triangles. Five amino acid positions on the DENV NS1 proteins with high frequency of detection of phosphorylated ions are indicated by blue arrows.

To determine the phosphorylation of DENV NS1, protein bands of cell-associated and extracellular NS1 (approximately 20–25 µg) from different cell origins were excised from the gels and processed for LC-MS/MS analysis of phosphopeptides. The mean percentage of protein sequence coverage identified by LC-MS/MS from different cell types tested ranged from 36.2% to 46.9%, and from 40.3% to 53.7% for cell-associated and extracellular NS1 proteins, respectively (Table 1). Analysis of DENV proteins prepared from all cell types revealed potential phosphorylation sites at 37 amino acid positions of cell-associated NS1 proteins and at 25 amino acid positions of extracellular NS1 proteins (Table 2, Tables S2 and S3). Among these positions, 24 amino acid residues were found to be common for potential phosphorylation in both cell-associated and extracellular NS1 proteins (Table 2, Tables S2 and S3). The presence of phosphorylated ions (pS/T and pY) was detected at specific amino acid positions of DENV NS1 with varying frequency as evidenced by the total number of phosphopeptides identified from either cell-associated NS1 or extracellular NS1, or from both proteins derived from all cell types (Table 2). The frequency of detection of the DENV NS1 phosphopeptides in each cell type is described in more detail in Tables S2 and S3. The highest frequency of phosphorylated ion detection was observed at eight amino acid positions of DENV NS1—five residues (i.e., T27, T29, Y32, T230, and S233) on both the cell-associated and extracellular NS1 proteins, and three residues (i.e., T126, S128, and T132) found predominantly on the cell-associated NS1 proteins (Table 2, Tables S2 and S3). Further analysis of the amino acid similarity of the DENV NS1 proteins from all four DENV serotypes was performed by alignment of 638 protein sequences retrieved from the Virus Pathogen Database and Analysis Resource (ViPR; https://www.viprbrc.org, accessed on 14 November 2020) for DENV based on virus isolation from humans in Thailand during the years 2000–2020 (Figure 2). The results showed that T27, T29, Y32, T230, and S233 appeared to be conserved in all four serotypes of DENV NS1, whereas T126, S128, and T132 varied among different serotypes (Figure 2). 

**Figure 3:**
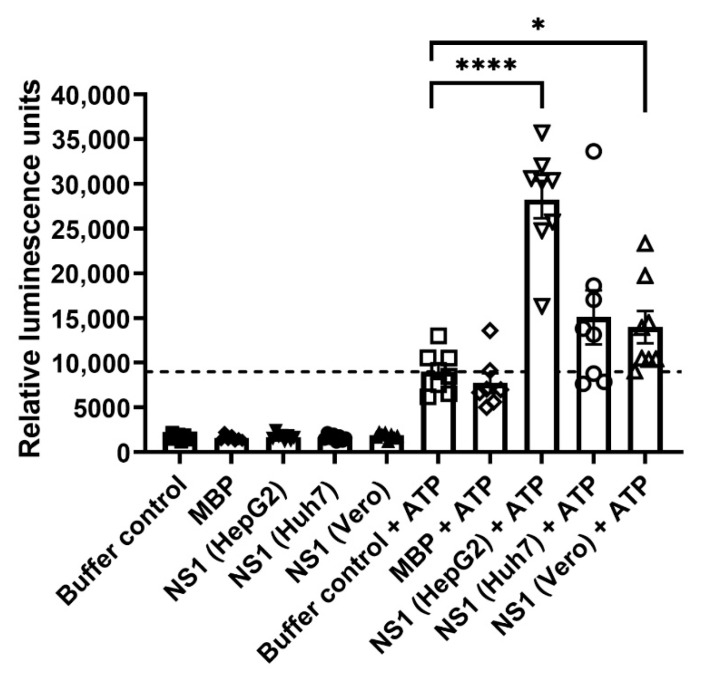
Kinase activity of DENV NS1 derived from different cell types. Extracellular NS1 proteins (0.5 µg) prepared from DENV-infected HepG2, Huh7, and Vero cell cultures were subjected to a kinase assay to determine their kinase activity in the presence or absence of ATP. Reactions that had no DENV NS1 (buffer control) or that contained myelin basic protein (MBP) served as a baseline control and an unrelated protein control (no kinase activity) in the assay, respectively. Kinase activity is presented in relative luminescence units. The results show the mean ± the standard error of the mean (SEM) of eight independent experiments. The dotted line represents the mean baseline level of buffer control in the presence of ATP. Differences in relative luminescence units between DENV NS1 derived from each cell type and buffer control in the presence of ATP were analyzed by an unpaired *t*-test (**** *p* < 0.0001, * *p* < 0.05).

A next set of experiments was conducted to assess by luminescence-based kinase assay whether the DENV NS1 proteins that were positive for the presence of phosphorylated ions possessed kinase activity. In the absence of ATP, the DENV NS1 proteins from HepG2, Huh7, and Vero cell cultures yielded low levels of relative luminescence similar to that of a kinase buffer control and MBP (an unrelated protein control) (Figure 3). An increased level of relative luminescence was observed in the kinase buffer control in the presence of ATP, which served as a baseline control for no kinase activity, and this level was comparable to that of the MBP control under the same condition (Figure 3). DENV NS1 proteins from three cell types exhibited differential levels of relative luminescence in the presence of ATP (indicative of kinase activity) that were greater than those of the baseline and MBP controls (Figure 3). The highest kinase activity was found in the HepG2-derived DENV NS1 protein (Figure 3). These findings suggest different degrees of kinase activity among DENV NS1 proteins obtained from different cell types. 

### 3.2. Effects of Phosphorylation Mutations on DENV Infection and NS1 Expression/Secretion

Five amino acid residues (i.e., T27, T29, Y32, T230, and S233) that are highly conserved among all four DENV serotypes (Figure 2) were found to be potential phosphorylation sites in both cell-associated and extracellular DENV NS1 proteins as evidenced by de novo peptide sequencing of LC-MS/MS (Figure S1). Consequently, these amino acid positions were selected for site-directed mutagenesis with alanine substitution to investigate the influence of phosphorylation mutations on DENV infection. Five NS1 mutations together with V6A (an unrelated control with decreased GRP78 association) were introduced into a Gibson assembly system to generate cDNA-derived infectious DENV as described in the Materials and Methods section, and the experimental strategies are also shown in Figure S2. We found that infectious DENV mutant with T29A, T230A, S233A, or V6A as well as wild-type virus could be generated from viral cDNA-transfected BHK-21 cell culture, whereas T27A and Y32A mutants were not recovered after BHK-21 transfection and two rounds of serial passages (P2) in Vero cell cultures (Figure S2). 

**Figure 4:**
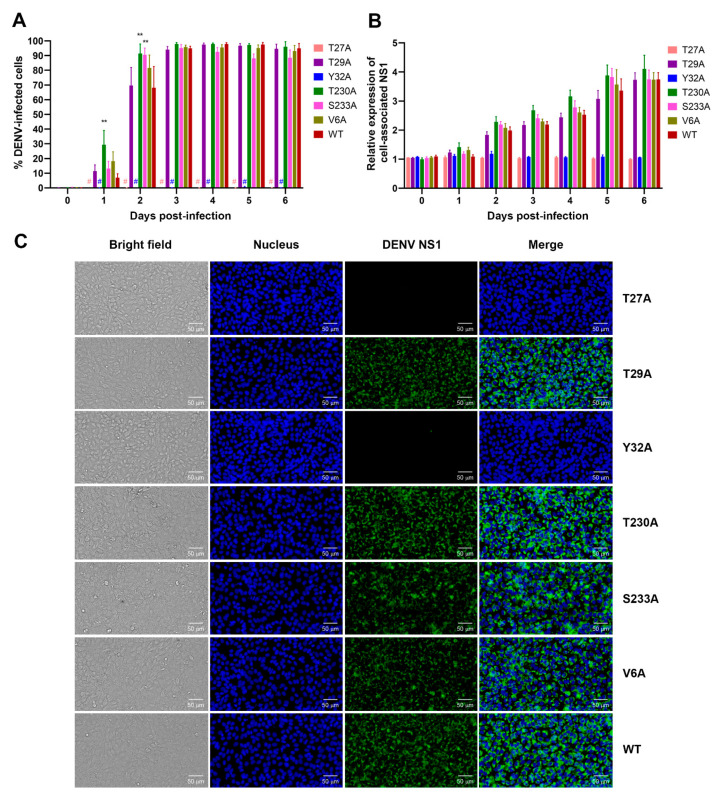
Effects of phosphorylation site mutations of DENV NS1 on DENV infection and NS1 expression. Vero cells were infected with wild-type DENV (WT) or different virus mutants (T27A, T29A, Y32A, T230A, S233A, or V6A). At varying time points post-infection, the infected cells were harvested and assessed for percentage of DENV-infected cells (**A**), for relative expression of cell-associated NS1 (**B**), and for the pattern of DENV NS1 expression (**C**) by immunofluorescence staining with a specific anti-NS1 antibody and high-content imaging and analysis. The results in (**A**) and (**B**) show the mean ± SEM of four independent experiments (** *p* < 0.05; #no detectable DENV-infected cells). Representative images of NS1-expressing cells are shown in (**C**).

To determine the effects of NS1 phosphorylation mutations on the kinetic profiles of DENV infection, Vero cells were infected with an equal amount of wild-type DENV or T29A, T230A, S233A, and V6A mutants (MOI 0.1), or with an equal volume of P2-derived culture supernatant of T27A and Y32A mutants for which infectious virus was undetectable. The virus-infected cells were assessed for the proportion, intensity, and pattern of DENV NS1 expression over time post-infection by immunofluorescence staining and high-content imaging and analysis. Cells expressing DENV NS1, which is a viral marker for DENV infection, were found to increase rapidly on days 1–2 and reached a plateau during days 3–6 after infection with T29A, T230A, S233A, and V6A mutants or their wild-type counterpart (Figure 4A). At the early time points post-infection, there were significantly higher percentages of DENV-infected cells detectable following infection with the T230A mutant (29% and 91% on days 1 and 2, respectively) and the S233A mutant (90% on day 2) as compared with wild-type virus infection (7% and 68% on days 1 and 2, respectively) (Figure 4A). However, similar percentages of DENV-infected cells (94%–98%) were found among the mutants (T29A, T230A, S233A, and V6A) and wild-type virus on day 3 post-infection, and there seemed to be no significant changes thereafter up to 6 days, except for a slight decrease in the percentage of DENV-infected cells observed at late time points after S233A infection (Figure 4A). Infection with the T29A, S233A, and V6A mutants and wild-type virus resulted in comparable levels of cell-associated NS1 expression at each time point tested (Figure 4B). Although infection with the T230A mutant was likely to yield a higher intensity of cell-associated NS1 expression than wild-type virus infection, the difference was not statistically significant (Figure 4B). In addition, these four mutants (T29A, T230A, S233A, and V6A) showed a perinuclear staining pattern of DENV NS1 expression in virus-infected cells similar to the wild-type virus (Figure 4C). Importantly, infection of Vero cells with P2-derived culture supernatants of the T27A and Y32A mutations did not show any detectable DENV-infected cells during the 6-day study period, thus providing further evidence of no active DENV replication in the cultures (Figure 4A–C). 

**Figure 5:**
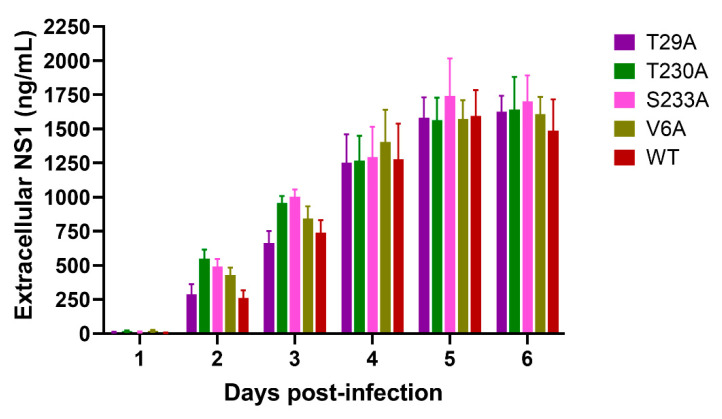
Effects of phosphorylation site mutations of DENV NS1 on secretion of DENV NS1 protein. Culture supernatants were collected from Vero cells infected with wild-type DENV or different virus mutants at the indicated time points post-infection and assayed for the levels of extracellular NS1 protein by NS1 ELISA. The results show the mean ± SEM of four independent experiments.

We next sought to determine the effects of phosphorylation mutations on DENV NS1 secretion by NS1 ELISA using supernatants collected from DENV-infected cell cultures. Extracellular NS1 proteins were detectable at low levels (5–19 ng/mL) on day 1, but levels increased more rapidly during days 2–4 after infection with the T29A, T230A, S233A, and V6A mutants and wild-type virus (Figure 5). This observation was consistent with the increased percentages of DENV-infected cells found during the same time period (Figure 4A). Even though the percentages of DENV-infected cells reached the maximum levels as early as day 3 post-infection (Figure 4A), extracellular NS1 protein levels were observed to plateau later on days 5–6 post-infection (Figure 5). There was no statistically significant difference in the detectable levels of extracellular NS1 proteins between the mutant (T29A, T230A, S233A, or V6A) and wild-type virus-infected cell cultures at all time points tested (Figure 5). 

**Figure 6:**
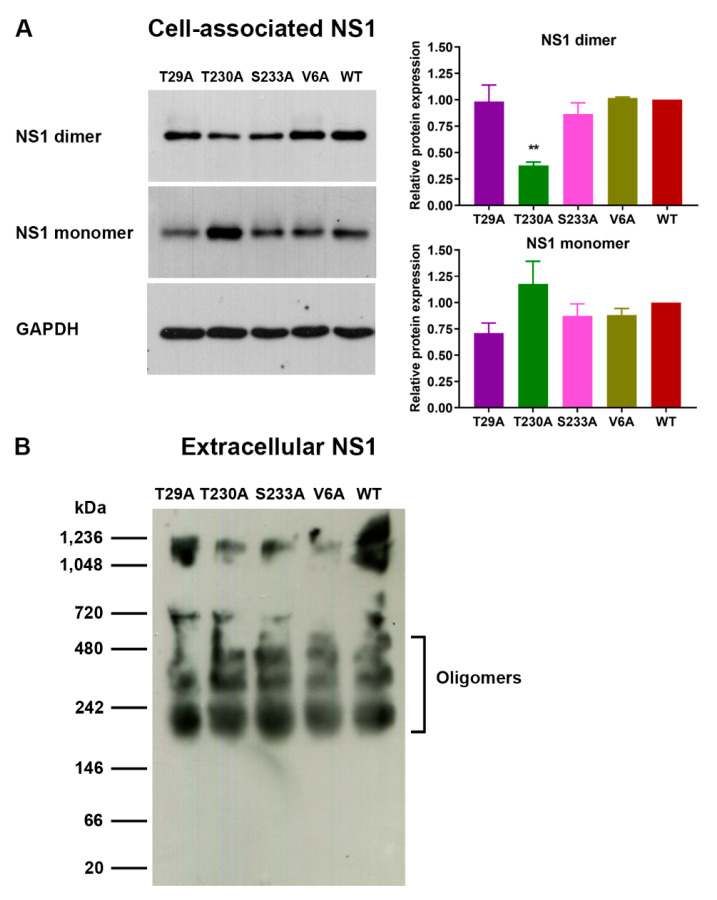
Effects of phosphorylation site mutations of DENV NS1 on the formation of cell-associated and extracellular NS1 proteins. Cell lysates and culture supernatants were harvested from Vero cells infected with wild-type DENV or different virus mutants at day 7 post-infection. (**A**) Cell lysates were treated with β-mercaptoethanol at 95 °C for 5 min or left untreated, and then subjected to 10% SDS-PAGE and subsequent immunoblotting to detect DENV NS1 monomer and human GAPDH (internal control) or DENV NS1 dimer, respectively. The representative results of three independent experiments with a similar outcome are shown in the **left panel**. Relative protein expression levels of DENV NS1 monomer and dimer was determined based on the normalization of protein band intensities to the internal control and are shown as the mean ± SEM of three independent experiments (**right panel**; ** *p* < 0.01). (**B**) Culture supernatants were subjected to native PAGE and immunoblotting analysis for DENV NS1 oligomers.

Since DENV NS1 proteins exist in different forms (monomer, dimer, and hexamer) in virus-infected cell culture [15,16,19,66], we further investigated whether phosphorylation mutations had any effect on DENV NS1 formation. SDS-PAGE and immunoblotting analysis of DENV-infected cell lysates was performed under either heated and reduced condition, or non-heated and non-reduced condition for the detection of a monomeric or dimeric form of the DENV NS1 protein, respectively. Both DENV NS1 monomer and dimer (approximately 48 kDa and 80 kDa, respectively) could be detected in lysates of Vero cells infected with T29A, T230A, S233A, and V6A mutants and wild-type virus (Figure 6A, left panel, and Figure S3A,B). Analysis of relative levels of cell-associated NS1 proteins by normalization of band intensity with human GAPDH (an internal control; 37 kDa) demonstrated that T230A mutant-infected cell lysate had significantly lower levels of NS1 dimer but relatively higher levels of NS1 monomer than wild-type virus-infected cell control, whereas no difference in the relative levels of DENV NS1 monomer and dimer was detected between cell lysates infected with other mutants (T29A, S233A, and V6A) and wild-type virus (Figure 6A, left and right panels, and Figure S3). In parallel, analysis of supernatants from virus-infected cell cultures by native PAGE and subsequent immunoblotting revealed the presence of multiple oligomeric forms (slightly smaller than 242 kDa and higher order) of the DENV NS1 proteins in a pattern that was similar among T29A, T230A, S233A, and V6A mutants and wild-type virus (Figure 6B). These oligomeric forms were consistent with the results from previously described reports that showed multiple bands of DENV NS1 oligomers ranging from 150 to 660 kDa using native PAGE and immunoblotting, and that estimated the molecular mass of hexameric NS1 between 250 and 350 kDa using size-exclusion chromatography [19,33,66,67]. Taken together, these findings indicate that specific mutations may affect the intrinsic property of DENV NS1 formation.

### 3.3. Effects of Phosphorylation Mutations on DENV Production

**Figure 7:**
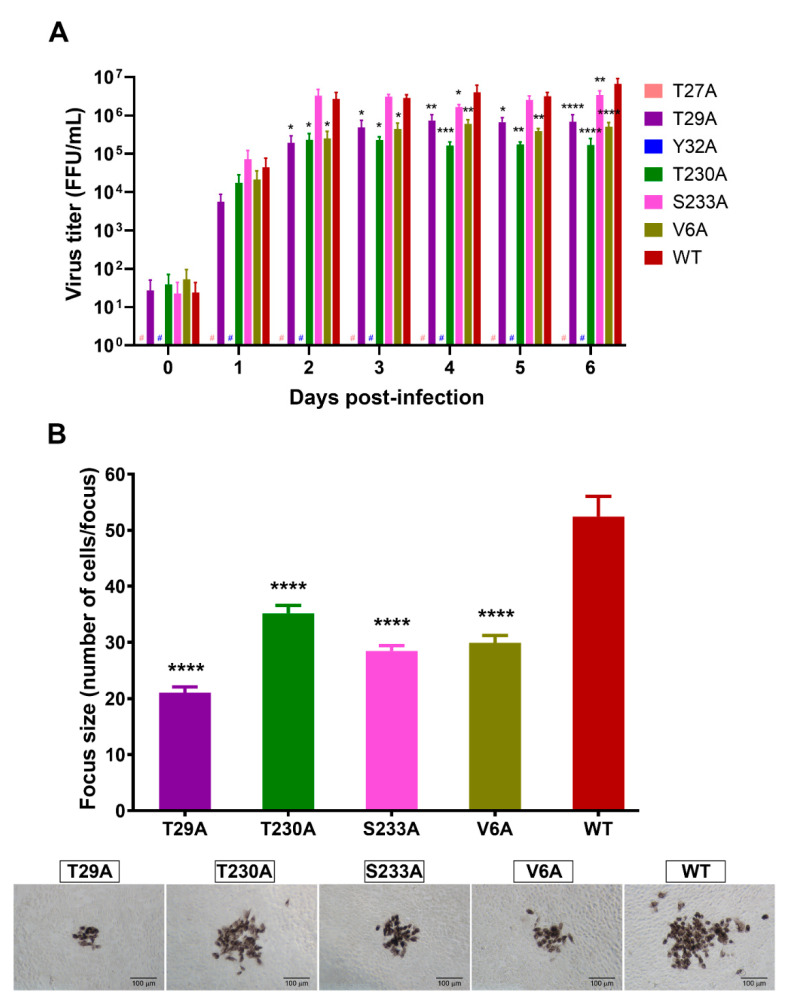
Effects of phosphorylation site mutations of DENV NS1 on DENV production and virus-infected cell foci. Vero cells were infected with wild-type DENV or different virus mutants, and culture supernatants were collected at the indicated time points post-infection to determine infectious DENV by FFU assay. (**A**) Production of infectious DENV in virus-infected cultures was reported as virus titers (FFU/mL). (**B**) The size of virus-infected cell foci following infection with wild-type DENV or different virus mutants was enumerated based on the number of DENV E-expressing cells per focus in the FFU assay. Approximately 101–134 virus-infected cell foci were analyzed, and their representative images are shown (bottom panel). The results in (**A**,**B**) demonstrate the mean ± SEM of four independent experiments (* *p* < 0.05; ** *p* < 0.01; *** *p* < 0.001; **** *p* < 0.0001; #no detectable DENV titers).

To address whether specific mutations at potential phosphorylation sites of NS1 have any effect on the production of infectious DENV, Vero cells were infected with DENV NS1 mutants or wild-type virus as described earlier, and culture supernatants were harvested daily for 6 days and subjected to a DENV titration assay. T27A and Y32A mutations resulted in no detectable DENV in culture supernatants at all time points tested (Figure 7). This was in accordance with the results of immunofluorescence staining assay that showed neither detectable DENV-infected cells nor DENV NS1 expression in the virus-infected cell cultures (Figure 4A,B). Rapid increases in virus production were detected within the first 2 days after infection with NS1 phosphorylation mutants (T29A, T230A, and S233A), an unrelated mutant (V6A), and wild-type virus (Figure 7A), and their virus titers plateaued at day 3 (Figure 7A), which is the same period at which the maximum number of DENV-infected cells was observed (Figure 4A). The T29A, T230A, and V6A mutants were detectable at significantly lower infectious virus titers than wild-type virus for almost the entire study period (Figure 7A). Unlike other mutants, the S233A mutant had titers of virus production similar to those of the wild-type virus during the early phase after infection (Figure 7A). Nevertheless, a tendency to detect lower titers of the S233A mutant was also discernable at certain late time points post-infection (Figure 7A). Further analysis of the focus size of virus-infected cells was performed to determine the characteristics of the infectious viruses. All four mutants (T29A, T230A, S233A, and V6A) produced significantly smaller foci of virus-infected cells than their wild-type counterpart (Figure 7B, top and bottom panels). 

**Figure 8:**
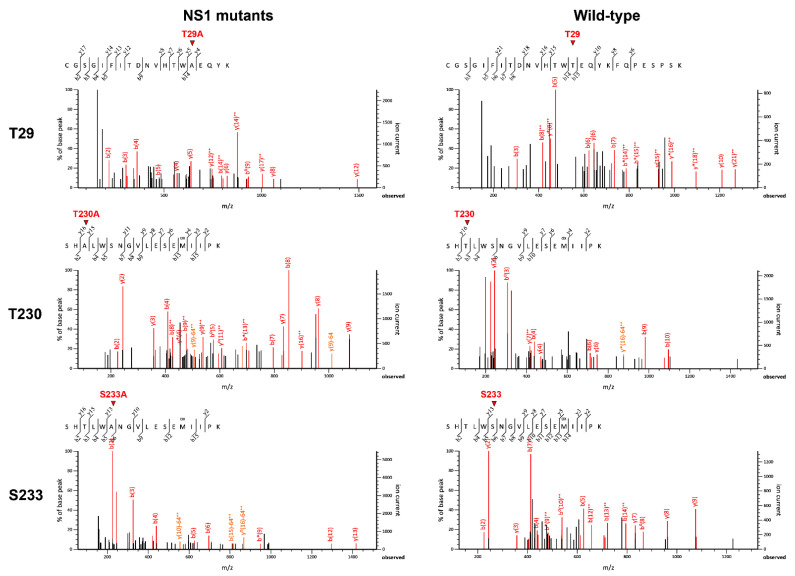
Verification of DENV NS1 phosphorylation mutations by LC-MS/MS. Lysates of Vero cells infected with wild-type DENV or different virus mutants at day 7 post-infection were processed for immunoprecipitation with anti-NS1 antibodies. The immunoprecipitated samples were analyzed by SDS-PAGE and Coomassie Brilliant Blue staining. DENV NS1 protein bands were excised from the gels and further processed for LC-MS/MS to verify the phosphorylation mutations at specific amino acid positions (T29A, T230A, and S233A) of DENV NS1. Three independent experiments were conducted. Representative results of de novo peptide sequencing show the substitution of Thr29, Thr230, and Ser233 with alanine in DENV NS1 protein derived from T29A, T230A, and S233A mutants (**left panel**) as compared with that from wild-type DENV (**right panel**).

To verify whether the effects on DENV production and characteristics stem from the mutations of NS1 phosphorylation sites, DENV NS1 proteins were immunoprecipitated from lysates of cells infected with the NS1 phosphorylation mutants (T29A, T230A, and S233A) and wild-type virus control and then subjected to LC-MS/MS analysis for de novo peptide sequencing. Results from three independent sets of experiments demonstrated that DENV NS1 proteins derived from the T29A, T230A, and S233A mutants contained alanine at the corresponding mutation sites (Figure 8, left panel), whereas those derived from their wild-type counterpart contained threonine at the amino acid positions 29 and 230, and serine at the amino acid position 233 (Figure 8, right panel). These findings confirmed the importance of specific amino acid residues of DENV NS1 to infectious DENV production.

## 4. Discussion

Viruses hijack host cellular machinery for their replication and survival in the host. Phosphorylation is an important post-translational modification process of host cells that regulates protein activity and stability, the subcellular localization of proteins, and protein–protein interactions [41,42,43]. Certain viral proteins undergo phosphorylation modifications in host cells in order to exert specific functions [68,69,70]. Previous studies have shown that flaviviruses utilize the host kinase machinery for the phosphorylation of NS5 protein (the most conserved protein) in cells infected with DENV and other viruses in the same family, as well as in cell-free systems [71,72,73,74]. The DENV NS5 protein functions in viral RNA replication via methyltransferase and RNA-dependent-RNA polymerase activities, and it also serves as a type I IFN antagonist by binding to signal transducer and activator of transcription 2 (STAT2) and subsequently blocking its phosphorylation [75,76,77]. The phosphorylation status of NS5 also correlates with subcellular localization and interaction with DENV NS3 protease required for viral polyprotein processing in host cells [71]. To our knowledge, there has been no report on the phosphorylation of other DENV proteins until now. Our study provides important evidence to demonstrate that the NS1 protein of DENV is potentially phosphorylated, and that its specific amino acid residues have significant effects on DENV production. 

Using LC-MS/MS analysis, we identified 24 potential phosphorylation sites on both cell-associated and extracellular NS1 proteins from three mammalian cell lines infected with DENV, and some of them (T27, T29, Y32, T230, and T233) were detectable at high frequency and are highly conserved among all four serotypes of DENV (Figure 2 and Table 2, Tables S2 and S3). Site-directed mutagenesis of these potential phosphorylation sites differentially affected the intrinsic property of DENV NS1 and the production of infectious viruses. Alanine substitution at either T27 or Y32 caused a deleterious effect on DENV infection, since no detectable virus was observed following the transfection of BHK-21 cell cultures with the mutant cDNA in two independent sets of experiments (Figure S2). Subsequent transfers of BHK-21 culture supernatants into Vero cell cultures showed a similar lack of virus production even after 14 days of culture (Figure S2). In addition, the kinetic study of DENV infection using the second passage (P2) of Vero cell-derived culture supernatants did not yield any recoverable virus in the T27A or Y32A-infected cultures (Figure 4A and Figure 7A). Therefore, our findings indicate the importance of the potentially phosphorylated DENV NS1 residues T27 and Y32 on the propagation of infectious viruses in host cells. Consistent with these results, a previous study in alanine substitutions of DENV NS1 in a DENV replicon system revealed a severe defect of Y32A mutant in viral RNA replication and infectious virus production and its impairment in NS1 and NS4A-2K-4B interaction [25,28]. 

Unlike the T27A and Y32A mutations, alanine substitution at T29, T230, or S233 resulted in the recovery of infectious DENV in the transfected BHK-21 cell cultures (Figure S2). These mutants were confirmed to possess the corresponding mutation sites on the DENV NS1 protein by LC-MS/MS analysis (Figure 8). A detailed kinetic study of T29A, T230A, and S233A mutants in Vero cell cultures demonstrated similar plateaus of percentage of DENV-infected cells, cell-associated NS1 expression, and extracellular NS1 levels as compared with wild-type DENV (Figure 4 and Figure 5); however, their titers in the culture supernatants were significantly lower than those of their wild-type counterpart (Figure 7A). All of the virus mutants also produced small focus sizes of DENV-infected cells, which is a phenotypic characteristic of virus attenuation (Figure 7B). Therefore, our findings suggest the important roles of T29, T230, and, to a lesser extent, S233 in DENV production possibly at the steps of the assembly and/or release of infectious virus particles. Furthermore, T230A mutation, but not T29A and S233A mutations, led to a significant decrease in the relative level of DENV NS1 dimer (Figure 6A), whereas none of these mutations affected DENV NS1 oligomeric formation (Figure 6B). The reduction in relative level of DENV NS1 dimer suggests that the T230A mutation interferes in dimer formation and/or dimer stability, which may in turn influence the production of infectious virus. As expected, the V6A mutant (unrelated control of phosphorylation), which contained an alanine substitution at a presumed site for NS1 association with human chaperone GRP78 [55], also exhibited a kinetic reduction of virus titers in the culture supernatants with an attenuation characteristic (Figure 7A,B) most likely owing to the impaired effect of NS1 and GRP78 interplay on DENV replication. 

Five amino acid residues (T27, T29, Y32, T230, and S233) with potential phosphorylation identified in our study are located in different domains (i.e., β-roll (amino acids 1–29), wing (amino acids 30–180), and β-ladder (amino acids 181–352)) of DENV NS1 according to the previously reported protein structure [78]. The β-roll is a hydrophobic dimerization domain and together with a greasy finger loop (amino acids 159–162) forms a hydrophobic protrusion on the NS1 structure that likely takes part in dimeric NS1 association with the host ER membrane and viral replication complex [78,79]. The binding of the NS1 protein with the viral NS4A-2K-4B precursor associated with the host membrane is also indispensable for genomic RNA replication of DENV [25]. Therefore, it might be possible that the T27, T29, and Y32 residues in the β-roll and a connector of the wing domain of DENV NS1 may play important roles in these viral and host interactions that provide a favorable platform to facilitate the amplification process of viral RNA. Whether the phosphorylation of these residues is a prerequisite for ER membrane and viral replication complex interactions of DENV NS1 (or vice versa) still needs to be addressed. In addition to viral RNA replication, the DENV NS1 protein has been proposed to have another function in the assembly of infectious virus particles via its binding with viral structural proteins (C, prM, and E) [28]. Some specific amino acid residues in the wing and β-ladder domains, such as S114 and T301, which were also identified in this study as potential phosphorylation sites (Table 2, Tables S2 and S3), were found to be critical for viral structural protein association [28]. Nevertheless, the function of the identified potential phosphorylation sites on the NS1 protein other than those five amino acid residues with alanine substitution was not defined in this study and should be further investigated. 

In this study, our kinase assays revealed varying degrees of kinase activity among DENV NS1 proteins purified from culture supernatants of different cell origins. These results suggest the possibility that the NS1 protein has intrinsic kinase activity and can autophosphorylate. Differences in the kinase activity of DENV NS1 proteins from various sources may be due to differential protein phosphorylation occurring in different cell types, as previously reported by other studies [80,81,82]. However, the notion that a host kinase(s) potentially co-purifying with DENV NS1 might also be responsible for the kinase activity in the reactions could not be ruled out. Important questions still need to be answered regarding which host protein kinases are responsible for the phosphorylation of DENV NS1 and whether the DENV NS1 protein can modulate any downstream process of host phosphorylation events. Since DENV NS1 may contain phosphorylation motifs that are targets of specific host kinases, we performed an analysis of DENV-2 NS1 amino acid sequence to search for potential functional linear motifs using the Eukaryotic Linear Motif resource (ELM, http://elm.eu.org/ accessed on 8 February 2021). We found predicted linear phosphorylation motifs—some of which were identified in our study by LC-MS/MS analysis—that could be recognized by human proteins, such as casein kinases (CK1 and CK2), glycogen synthase kinase 3 (GSK3), MAPK-interacting molecules, NIMA-related kinase 2 (NEK2), STAT3, polo-like kinases (PLK), and phosphatidylinositol 3-kinase-related kinases (PIKK) (Table S4). Specifically, amino acid positions 32–35 of DENV NS1 (YKFQ) encompass a motif (YXXQ) similar to that found in the cytoplasmic region of cytokine receptors that bind to the STAT3 SH2 domain (Table S4). Consistent with that, a previous study using a yeast two-hybrid system with a human bone-marrow cDNA library demonstrated the interaction of cell-associated DENV NS1 with STAT3β, which might contribute to the pathogenesis of dengue [48]. Amino acid positions 103, 113, and 114, which are identified as potential phosphorylation sites of the DENV NS1 protein (Table 2, Tables S2, and S3), were found to be within linear motifs of GKRSLR (positions 100–105) and LKYSWK (positions 111–116), which were predicted for recognition by the human NEK2 protein based on the ELM analysis (Table S4). This observation is in line with our previous study that showed an association between the DENV NS1 and NEK2 proteins in virus-infected cells [53]. In that same study using immunoprecipitation and LC-MS/MS analysis, we also identified other human serine/threonine kinases and phosphatases that potentially interact with DENV NS1 during DENV infection. Additionally, DENV NS1 has been shown to activate the p38 MAPK pathway in endothelial cells, and this cellular pathway plays important roles in vascular permeability, apoptosis, and inflammatory response, and it is required for DENV infection [40,44]. Therefore, the relationship between DENV NS1 and its associated proteins should be studied in more detail to understand the mechanisms of NS1 phosphorylation and its downstream effects on host cellular events that might regulate DENV infection and dengue pathogenesis in host cells.

## 5. Conclusions

This study demonstrates for the first time the potential phosphorylation of the DENV NS1 protein following DENV infection. Five identified phosphorylation sites, all of which are highly conserved among all four serotypes of DENV, contribute differentially to the intrinsic property of the NS1 protein and infectious DENV production. The knowledge will be useful for future investigation of the host phosphorylation machinery hijacked by the DENV NS1 protein and for the potential development of target-specific antiviral drug design.

## Figures and Tables

**Table 1:** Sequence coverage of the DENV NS1 protein as analyzed by LC-MS/MS ^1^.

Sample	% Sequence Coverage of Cell-Associated NS1		
	HepG2	Huh7	Vero
1.1	32	35	15
1.2	47	24	21
1.3	42	49	12
2.1	49	67	40
2.2	50	46	40
2.3	51	59	35
3.1	40	17	65
3.2	57	10	56
3.3	54	19	69
Average	46.9	36.2	39.2
**Sample**	**% Sequence Coverage of Extracellular NS1**		
	**HepG2**	**Huh7**	**Vero**
1.1	48	44	54
1.2	45	42	58
1.3	28	50	49
Average	40.3	45.3	53.7

^1^ Cell-associated or extracellular NS1 proteins were purified from HepG2, Huh7, or Vero cell cultures infected with DENV. There were three independent sets of cell-associated NS1 proteins and one set of extracellular NS1 proteins for each cell type. Each set of samples was injected three times into LC-MS/MS for phosphopeptide analysis. Identified peptides were analyzed for amino acid sequence coverage of DENV NS1 by Mascot software.

**Table 2:** Detection of potential phosphorylation sites at specific amino acid positions on cell-associated and extracellular NS1 proteins from three cell types ^1^.

DENV NS1		The Number of Phosphopeptides Identified from Cell-Associated NS1 ^2^	The Number of Phosphopeptides Identified from Extracellular NS1 ^3^	Total Number of Identified Phosphopeptides ^4^
Position	Amino Acid			
17	S	1	1	2
22	T	2	3	5
27	T	9	10	19
29	T	13	6	19
32	Y	11	8	19
59	S	0	1	1
61	T	1	0	1
72	T	1	0	1
80	S	2	0	2
87	T	3	3	6
90	T	5	2	7
103	S	3	3	6
113	Y	4	0	4
114	S	3	1	4
117	T	6	1	7
125	S	8	1	9
126	T	13	1	14
128	S	12	1	13
132	T	11	1	12
181	S	3	1	4
185	S	3	0	3
200	Y	1	0	1
204	S	1	0	1
216	S	1	0	1
228	S	1	0	1
230	T	14	11	25
233	S	14	13	27
239	S	5	1	6
252	S	2	1	3
256	Y	1	0	1
262	T	1	0	1
265	T	1	0	1
297	S	2	3	5
300	T	1	3	4
301	T	4	4	8
302	T	2	2	4
304	S	2	3	5
309	T	1	0	1

^1^ Three independent sets of cell-associated NS1 proteins and one set of extracellular NS1 proteins were prepared from HepG2, Huh7, and Vero cell cultures infected with DENV. Each set of samples was injected three times into LC-MS/MS for phosphopeptide analysis. Frequencies of detection of phosphorylated ions in all cell types were analyzed from all LC-MS/MS injections. ^2,3,4^ Data show the number of phosphopeptides detected in cell-associated NS1, extracellular NS1, or both samples, respectively, prepared from all cell types.

## Supplementary Materials

The following are available online at https://www.mdpi.com/article/10.3390/v13071393/s1, Figure S1: Detection of phosphorylated ions at specific amino acid residues of DENV NS1 protein, Figure S2: Generation of infectious DENV with NS1 mutations at potential phosphorylation sites, Figure S3: Immunoblotting analysis of cell-associated DENV NS1 proteins in wild-type DENV and mutant-infected cell cultures, Table S1: Oligonucleotide primer sequences used for site-directed mutagenesis of DENV NS1 by alanine substitution at specific amino acid positions, Table S2: Frequency of detection of potential phosphorylation sites at specific amino acid positions of the cell-associated NS1 protein, Table S3: Frequency of detection of potential phosphorylation sites at specific amino acid positions of the extracellular NS1 protein, Table S4: Analysis of functional linear motifs of DENV NS1 using the Eukaryotic Linear Motif (ELM) resource.
