# Supplementary material for: Potential Phosphorylation of Viral Nonstructural Protein 1 in Dengue Virus Infection
Source: Viruses. 2021 Jul 17;13(7):1393. doi: 10.3390/v13071393 (PMC8310366; doi:10.3390/v13071393)
Supplement: Supplementary file 1 [file viruses-13-01393-s001.zip › Supplementary Figure S1.pdf]

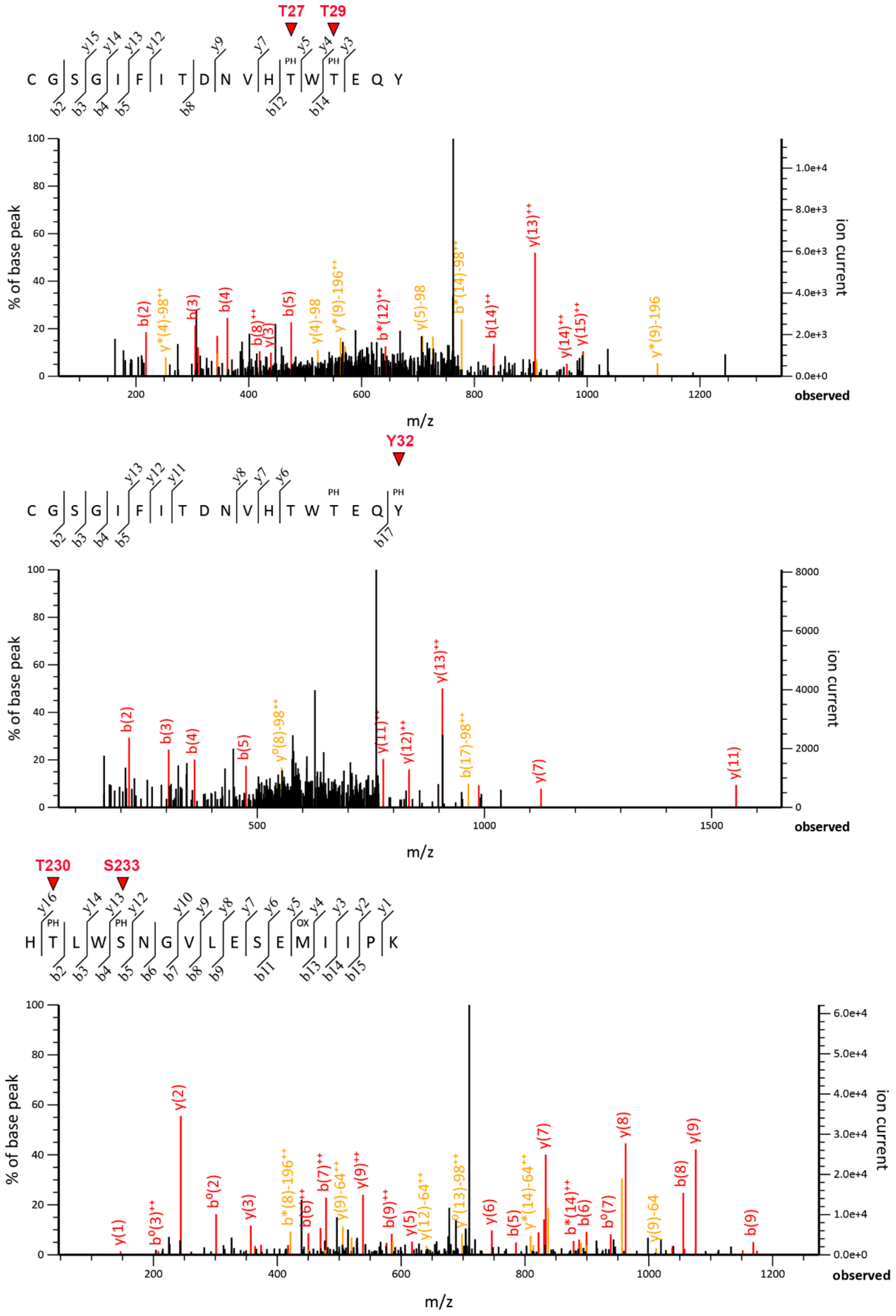
T27
T29
C G S G I F I T D N V H T W T E Q Y
PH
% of base peak
ion current
m/z
observed
b(2)
y*(4)-98++
b(3)
b(4)
b(8)++
y(3)
b(5)
y(4)-98
y*(9)-196++
b*(12)++
y(5)-98
b*(14)-98++
b(14)++
y(13)++
y(14)++
y(15)++
y*(9)-196
Y32
C G S G I F I T D N V H T W T E Q Y
b(2)
b(3)
b(4)
b(5)
yº(8)-98++
y(11)++
y(12)++
y(13)++
b(17)-98++
y(7)
y(11)
T230
S233
H T L W S N G V L E S E M I I P K
OX
y(1)
bº(3)++
y(2)
bº(2)
y(3)
b*(8)-196++
b(6)
b(7)++
y(9)-64++
y(9)++
b(9)++
y(5)
y(12)-64++
yº(13)-98++
y(6)
b(5)
y*(14)-64++
y(7)
b*(14)++
b(6)
bº(7)
y(8)
y(9)-64
b(8)
y(9)
b(9)

Figure S1. Detection of phosphorylated ions at specific amino acid residues of DENV NS1 protein. Cell-associated NS1 and extracellular NS1 proteins from HepG2, Huh7, and Vero cells infected with DENV-2 were processed for LC-MS/MS to determine potential phosphorylation sites. Tryptic peptides were analyzed for the presence of phosphorylated ions (pS/T and pY) at specific amino acid positions of the DENV NS1 protein by de novo peptide sequencing. Representative results show detection of phosphorylated ions at Thr27, Thr29, Tyr32, Thr230, and Ser233 (red triangles) of the DENV NS1 protein.
