# Supplementary material for: Potential Phosphorylation of Viral Nonstructural Protein 1 in Dengue Virus Infection
Source: Viruses. 2021 Jul 17;13(7):1393. doi: 10.3390/v13071393 (PMC8310366; doi:10.3390/v13071393)
Supplement: Supplementary file 1 [file viruses-13-01393-s001.zip › Supplementary Figure S2.pdf]

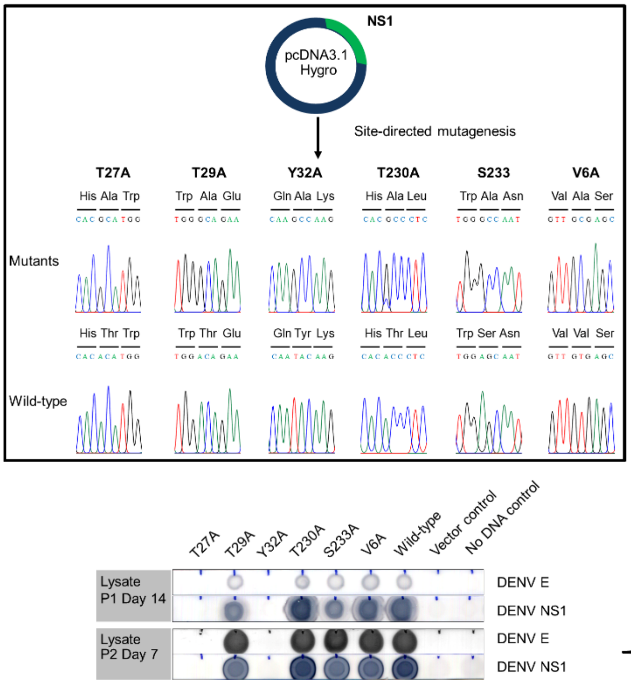

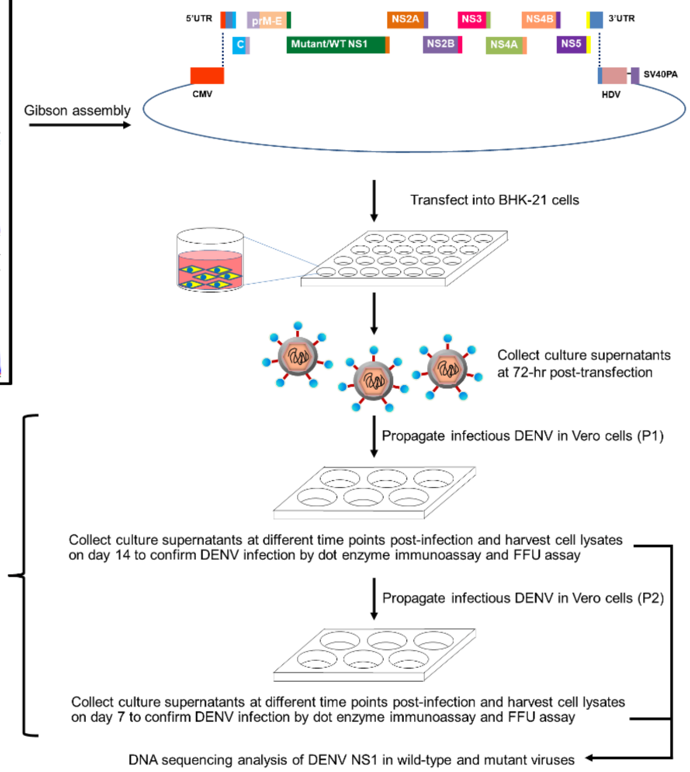

	Virus Titer (FFU/ml)			
	P1 day 11	P1 day 14	P2 day 4	P2 day 7
T27A	0.00E+00	0.00E+00	0.00E+00	0.00E+00
T29A	2.90E+04	9.55E+04	6.82E+04	7.27E+04
Y32A	0.00E+00	0.00E+00	0.00E+00	0.00E+00
T230A	4.55E+04	4.09E+04	1.05E+05	5.00E+04
S233A	4.55E+04	1.05E+05	2.91E+05	4.41E+05
V6A	4.55E+04	1.23E+05	4.55E+04	5.00E+04
Wild-type	1.45E+05	1.68E+05	2.90E+05	2.59E+05
Vector control	0.00E+00	0.00E+00	0.00E+00	0.00E+00
No DNA control	0.00E+00	0.00E+00	0.00E+00	0.00E+00

Figure S2. Generation of infectious DENV with NS1 mutations at potential phosphorylation sites. pcDNA-Hygro plasmid constructs carrying wild-type DENV NS1 or different DENV NS1 mutants (T27A, T29A, Y32A, T230A, S233A, or V6A) were used as templates for amplification of DENV NS1-coding amplicons. The corresponding NS1 fragments together with other DENV gene fragments and a plasmid expression vector were assembled into DENV infectious DNA by Gibson assembly, and the resulting DENV DNA products were transfected into BHK-21 cells using Lipofectamine 2000. At day 3 post-transfection, culture supernatants were harvested and transferred into Vero cell cultures to propagate the infectious DENV. Culture supernatants and cell lysates were collected at different time points post-infection and assessed for the presence of infectious DENV by dot enzyme immunoassay with anti-DENV E and anti-NS1 specific antibodies and FFU assay. Two rounds of DENV propagation (P1 and P2) were performed in Vero cell cultures. DENV NS1 sequences of wild-type and mutant viruses were verified by DNA sequencing analysis.
