# Supplementary material for: Potential Phosphorylation of Viral Nonstructural Protein 1 in Dengue Virus Infection
Source: Viruses. 2021 Jul 17;13(7):1393. doi: 10.3390/v13071393 (PMC8310366; doi:10.3390/v13071393)
Supplement: Supplementary file 1 [file viruses-13-01393-s001.zip › Supplementary Figure S3.pdf]

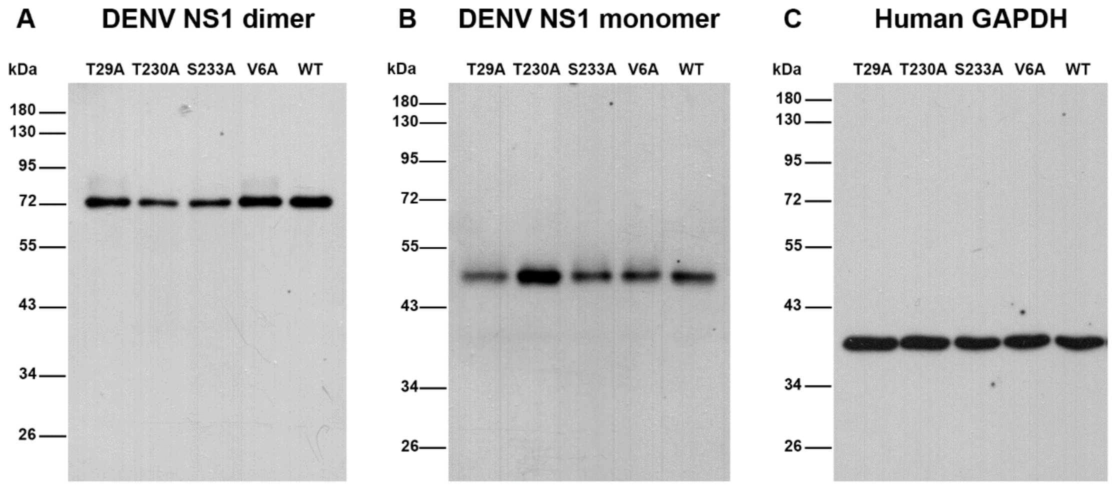

Figure S3. Immunoblotting analysis of cell-associated DENV NS1 proteins in wild-type DENV and mutant-infected cell cultures. Cell lysates were harvested from Vero cells infected with wild-type DENV or different virus mutants at day 7 post-infection. The lysate samples were untreated or treated with β-mercaptoethanol at 95 °C for 5 minutes, and then subjected to 10% SDS-PAGE and subsequent immunoblotting using specific antibodies to detect DENV NS1 dimer (**A**), or DENV NS1 monomer and human GAPDH (internal control) (**B** and **C**), respectively. The representative results of three independent experiments with a similar outcome are shown.
