# Supplementary material for: Potential Phosphorylation of Viral Nonstructural Protein 1 in Dengue Virus Infection
Source: Viruses. 2021 Jul 17;13(7):1393. doi: 10.3390/v13071393 (PMC8310366; doi:10.3390/v13071393)
Supplement: Supplementary file 1 [file viruses-13-01393-s001.zip › Supplementary Table S1.pdf]

**Table S1. Oligonucleotide primer sequences used for site-directed mutagenesis of DENV NS1 by alanine substitution at specific amino acid positions**

| DENV NS1 mutation | Oligonucleotide primer sequences |
|---|---|
| T27A | Forward: 5'-AACGTGCACGCATGGACAGAACAATACAAG-3' |
| | Reverse: 5'-TGTCCATGCGTGCACGTTGTCTGTGATGTT-3' |
| T29A | Forward: 5'-CACACATGGGCAGAACAATACAAGTTCCAA-3' |
| | Reverse: 5'-TTGTTCTGCCCATGTGTGCACGTTGTC-3' |
| Y32A | Forward: 5'-GCACACATGGACAGAACAAGCCAAGTTCCAACCAGAATCC-3' |
| | Reverse: 5'-GGATTCTGGTTGGAACTTGGCTTGTTCTGTCCATGTGTGC-3' |
| T230A | Forward: 5'-AAATCACACGCCCTCTGGAGCAATGGAGTG-3' |
| | Reverse: 5'-CCAGAGGGCGTGTGATTTTGGCCAGTGGCA-3' |
| S233A | Forward: 5′-AAAATCACACACCCTCTGGGCCAATGGAGTGCTAGAAAGT-3′ |
| | Reverse: 5′-ACTTTCTAGCACTCCATTGGCCCAGAGGGTGTGTGATTTT-3′ |
| V6A | Forward: 5′- GGTTGCGTTGCGAGCTGGAAAAACAAAGAA-3′ |
| | Reverse: 5′-CCAGCTCGCAACGCAACCACTATCGGCCTG-3′ |
