# Supplementary material for: Potential Phosphorylation of Viral Nonstructural Protein 1 in Dengue Virus Infection
Source: Viruses. 2021 Jul 17;13(7):1393. doi: 10.3390/v13071393 (PMC8310366; doi:10.3390/v13071393)
Supplement: Supplementary file 1 [file viruses-13-01393-s001.zip › Supplementary Table S2.pdf]

Table S2. Frequency of detection of potential phosphorylation sites at specific amino acid positions of the cell-associated NS1 protein

Source		Intracellular NS1																														Overall frequency of detection of phosphopeptides
Cell type		HepG2									Sum	Huh7									Sum	Vero									Sum	
Sample set		1			2			3				1			2			3				1			2			3				
Position	Amino acid	1.1	1.2	1.3	2.1	2.2	2.3	3.1	3.2	3.3		1.1	1.2	1.3	2.1	2.2	2.3	3.1	3.2	3.3		1.1	1.2	1.3	2.1	2.2	2.3	3.1	3.2	3.3		
17	S	-	-	-	-	-	-	-	-	-	0	-	-	-	-	-	-	-	-	-	0	-	-	-	-	-	-	-	-	1	1	1
22	T	-	-	1	-	-	-	-	-	-	1	-	-	-	-	-	-	-	-	-	0	-	-	-	-	-	-	-	-	1	1	2
27	T	-	-	1	-	1	-	-	1	-	3	-	-	-	-	1	1	-	-	-	2	-	-	-	-	-	1	1	-	2	4	9
29	T	2	-	2	1	-	-	-	1	-	6	1	-	1	2	1	-	-	-	-	5	-	-	-	-	-		-	-	2	2	13
32	Y	2	-	-	1	1	-	-	-	-	4	1	-	1	2	-	1	-	-	-	5	-	-	-	-	-	1	1	-	-	2	11
59	S	-	-	-	-	-	-	-	-	-	0	-	-	-	-	-	-	-	-	-	0	-	-	-	-	-	-	-	-	-	0	0
61	T	-	-	-	-	-	-	-	-	-	0	-	-	-	-	-	-	-	-	-	0	-	1	-	-	-	-	-	-	-	1	1
72	T	-	-	-	-	-	1	-	-	-	1	-	-	-	-	-	-	-	-	-	0	-	-	-	-	-	-	-	-	-	0	1
80	S	-	-	-	-	-	1	-	1	-	2	-	-	-	-	-	-	-	-	-	0	-	-	-	-	-	-	-	-	-	0	2
87	T	-	1	-	-	-	-	-	1	-	2	-	-	-	-	-	-	-	-	-	0	-	-	-	-	-	1	-	-	-	1	3
90	T	-	1	-	-	-	-	-	1	1	3	-	-	-	-	-	-	1	-	-	1	-	-	-	-	-	1	-	-	-	1	5
103	S	-	-	-	-	1	-	-	1	-	2	-	-	-	-	-	-	1	-	-	1	-	-	-	-	-	-	-	-	-	0	3
113	Y	-	1	-	-	-	-	-	-	1	2	-	-	-	-	-	-	-	-	-	0	-	-	-	-	-	-	-	1	1	2	4
114	S	-	1	-	-	-	-	-	-	-	1	-	-	-	-	-	-	-	-	-	0	-	-	-	-	-	-	-	1	1	2	3
117	T	-	1	-	-	-	2	-	-	-	3	-	-		-	-	-	-	-	-	0	-	-	-	-	-	-	1	1	1	3	6
125	S	-	1	-	-	-	2	-	-	-	3	-	-	1	1	-	-	-	-	-	2	-	-	-	-	-	-	1	1	1	3	8
126	T	-	1	1	1	1	2	-	1	-	7	-	-	1	2	-	1	-	-	-	4	-	-	-	-	-	-	1	-	1	2	13
128	S	-	1	1	1	1	1	-	1	1	7	-	-	-	2	-	1	-	-	-	3	-	-	-	-	-	-	1	-	1	2	12
132	T	-	-	1	1	1	1	-	1	1	6	-	-	-	2	-	1	-	-	-	3	-	-	-	-	1	-	-	-	1	2	11
181	S	-	-	-	-	-	-	-	-	-	0	-	-	-	-	-	1	-	-	1	2	-	-	-	-	-	-	-	-	1	1	3
185	S	-	-	-	-	-	-	-	-	-	0	-	-	-	1	-	1	-	-	1	3	-	-	-	-	-	-	-	-	-	0	3
200	Y	-	-	-	-	-	-	-	-	-	0	-	-	-	-	-	-	-	-	-	0	-	-	-	-	1	-	-	-	-	1	1
204	S	-	-	-	-	-	-	-	-	-	0	-	-	-	-	-	-	-	-	-	0	-	-	-	-	1	-	-	-	-	1	1
216	S	-	-	-	-	-	-	-	1	-	1	-	-	-	-	-	-	-	-	-	0	-	-	-	-	-	-	-	-	-	0	1
228	S	-	-	-	-	-	-	-	-	-	0	-	-	-	-	-	-	-	-	-	0	-	-	-	-	-	-	1	-	-	1	1
230	T	-	-	-	-	1	1	1	-	1	4	-	-	-	-	2	1	1	-	1	5	-	-	-	-	1	1	2	1	-	5	14
233	S	-	-	-	-	1	1	1	1	1	5	-	-	-	-	2	1	1	-	1	5	-	-	-	-	1	1	1	1	-	4	14
239	S	-	-	-	-	1	-	-	-	-	1	-	-	-	1	-	-	-	-	1	2	-	-	-	-	-	-	1	-	1	2	5
252	S	-	-	-	-	-	-	-	-	-	0	-	-	-	-	-	-	-	-	-	0	-	-	-	1	-	-	-	-	1	2	2
256	Y	-	-	-	-	-	-	-	-	-	0	-	-	-	-	-	-	-	-	-	0	-	-	-	-	-	-	-	-	1	1	1
262	T	-	-	-	-	-	-	-	-	-	0	-	-	-	-	-	-	-	-	-	0	-	-	-	1	-	-	-	-	-	1	1
265	T	-	-	-	-	-	-	-	-	-	0	-	-	-	-	-	-	-	-	-	0	-	-	-	-	-	-	-	-	1	1	1
297	S	-	-	-	-	-	-	-	-	-	0	1	-	1	-	-	-	-	-	-	2	-	-	-	-	-	-	-	-	-	0	2
300	T	-	-	-	-	-	-	-	-	-	0	1	-	-	-	-	-	-	-	-	1	-	-	-	-	-	-	-	-	-	0	1
301	T	-	-	1	-	-	-	-	-	-	1	1	1	-	-	-	-	-	-	-	2	1	-	-	-	-	-	-	-	-	1	4
302	T	-	-	-	-	-	-	-	-	-	0	1	-	-	-	-	-	-	-	-	1	1	-	-	-	-	-	-	-	-	1	2
304	S	-	-	-	-	-	-	-	-	-	0	-	-	1	-	-	-	-	-	-	1	1	-	-	-	-	-	-	-	-	1	2
309	T	-	-	-	-	-	-	-	-	-	0	-	-	1	-	-	-	-	-	-	1	-	-	-	-	-	-	-	-	-	0	1
