# Supplementary material for: Potential Phosphorylation of Viral Nonstructural Protein 1 in Dengue Virus Infection
Source: Viruses. 2021 Jul 17;13(7):1393. doi: 10.3390/v13071393 (PMC8310366; doi:10.3390/v13071393)
Supplement: Supplementary file 1 [file viruses-13-01393-s001.zip › Supplementary Table S3.pdf]

Table S3. Frequency of detection of potential phosphorylation sites at specific amino acid positions of the extracellular NS1 protein

Source		Extracellular NS1												Overall frequency of detection of phosphopeptides
Cell type		HepG2			Sum	Huh7			Sum	Vero			Sum	
Sample set		1				1				1				
Position	Amino acid	2.1	2.2	2.3		3.1	3.2	3.3		1.1	1.2	1.3		
17	S	-	-	1	1	-	-	-	0	-	-	-	0	1
22	T	-	-	1	1	-	1	-	1	1	-	-	1	3
27	T	1	1	-	2	2	1	-	3	1	3	1	5	10
29	T	1	1	-	2	1	-	-	1	-	1	2	3	6
32	Y	-	-	-	0	1	2	-	3	2	2	1	5	8
59	S	-	-	1	1	-	-	-	0	-	-	-	0	1
61	T	-	-	-	0	-	-	-	0	-	-	-	0	0
72	T	-	-	-	0	-	-	-	0	-	-	-	0	0
80	S	-	-	-	0	-	-	-	0	-	-	-	0	0
87	T	-	-	1	1	1	1	-	2	-	-	-	0	3
90	T	-	-	1	1	-	-	-	0	-	1	-	1	2
103	S	-	-	-	0	-	-	1	1	1	1	-	2	3
113	Y	-	-	-	0	-	-	-	0	-	-	-	0	0
114	S	-	-	-	0	-	-	-	0	1	-	-	1	1
117	T	-	-	-	0	-	-	-	0	1	-	-	1	1
125	S	-	1	-	1	-	-	-	0	-	-	-	0	1
126	T	-	1	-	1	-	-	-	0	-	-	-	0	1
128	S	-	1	-	1	-	-	-	0	-	-	-	0	1
132	T	-	1	-	1	-	-	-	0	-	-	-	0	1
181	S	-	-	-	0	-	-	-	0	-	1	-	1	1
185	S	-	-	-	0	-	-	-	0	-	-	-	0	0
200	Y	-	-	-	0	-	-	-	0	-	-	-	0	0
204	S	-	-	-	0	-	-	-	0	-	-	-	0	0
216	S	-	-	-	0	-	-	-	0	-	-	-	0	0
228	S	-	-	-	0	-	-	-	0	-	-	-	0	0
230	T	1	2	1	4	2	1	-	3	1	2	1	4	11
233	S	1	2	2	5	2	1	-	3	1	2	2	5	13
239	S	-	-	-	0	-	-	-	0	-	1	-	1	1
252	S	-	1	-	1	-	-	-	0	-	-	-	0	1
256	Y	-	-	-	0	-	-	-	0	-	-	-	0	0
262	T	-	-	-	0	-	-	-	0	-	-	-	0	0
265	T	-	-	-	0	-	-	-	0	-	-	-	0	0
297	S	-	-	-	0	-	-	-	0	2	-	1	3	3
300	T	-	-	-	0	-	-	-	0	2	-	1	3	3
301	T	1	-	-	1	-	-	-	0	2	-	1	3	4
302	T	-	-	-	0	-	-	-	0	1	-	1	2	2
304	S	1	-	-	1	-	-	-	0	1	-	1	2	3
309	T	-	-	-	0	-	-	-	0	-	-	-	0	0
